# New antipsychotic drugs for the treatment of agitation and psychosis in Alzheimer’s disease: focus on brexpiprazole and pimavanserin

**DOI:** 10.12688/f1000research.22662.1

**Published:** 2020-07-08

**Authors:** Filippo Caraci, Mario Santagati, Giuseppe Caruso, Dario Cannavò, Gian Marco Leggio, Salvatore Salomone, Filippo Drago

**Affiliations:** 1 Department of Drug Sciences, University of Catania, Viale Andrea Doria 6, 95125, Catania, Italy; 2 Oasi Research Institute - IRCCS, Via Conte Ruggero 73, 94018, Troina, Italy; 3 ASP3 Catania, Department of Mental Health, Alzheimer Psychogeriatric Center Corso Italia 234, 95127, Catania, Italy; 4 Department of Biomedical and Biotechnological Sciences, University of Catania, Via S. Sofia 89, 95123, Catania, Italy

**Keywords:** Alzheimer’s disease, agitation, psychosis, second-generation antipsychotics, brexpiprazole, pimavanserin

## Abstract

Behavioral and psychological symptoms of dementia are symptoms of disturbed perception, mood, behavior, and thought content that occurred frequently. These symptoms, which include apathy, depression, anxiety, psychosis, agitation, and aggression, can serve as predictors of and early clinical diagnostic markers for Alzheimer’s disease (AD) and are common precipitants of institutional care. Agitation and psychosis are associated with accelerated disease progression and increased tau phosphorylation in patients with AD. Current guidelines recommend the use of second-generation antipsychotics for the treatment of agitation and psychosis in AD, but only after first-line non-pharmacological interventions and for no longer than 12 weeks because long-term use of these drugs is associated with an increased risk of mortality and an increased frequency of cerebrovascular events. Therefore, new antipsychotic drugs with improved efficacy and safety are needed as an alternative to current antipsychotic drugs. In this report, we discuss some of the most relevant advances in the field of agitation and psychosis in AD and focus on the recent positive clinical evidence observed with two new antipsychotics drugs: brexpiprazole and pimavanserin. Brexpiprazole is a receptor partial agonist (D2, D3, 5-HT1A), receptor antagonist (5-HT2A/B, α1B/α2C) according to the neuroscience-based nomenclature. Two recent phase III clinical trials have shown that brexpiprazole 2 mg/day is effective for the treatment of agitation in patients with AD and has an improved tolerability and safety profile compared with currently available second-generation antipsychotics. Pimavanserin is a receptor antagonist (5-HT2A, 5-HT2C) that has been given market authorization for psychosis occurring in Parkinson’s disease. Recent phase II studies suggest that this drug is effective in AD patients with more severe psychosis, although further long-term studies are needed to better define the efficacy and long-term safety profile of pimavanserin for the treatment of psychosis in AD.

## Behavioral and psychological symptoms in Alzheimer’s disease

Alzheimer’s disease (AD) is the most common form of dementia. Recent epidemiological data indicate that the number of people with AD worldwide will grow from the current 46.8 million to 131.5 million by 2050 ^1, 2^. AD is a neurodegenerative disorder characterized by memory deficit, cognitive decline, and neuropsychiatric symptoms, the so-called behavioral and psychological symptoms of dementia (BPSD) ^3^. According to the definition of the International Psychogeriatric Association, BPSD are “symptoms of disturbed perception, thought content, mood, and behavior frequently occurring in patients with dementia” ^3^. These symptoms include apathy, depression, anxiety, psychosis, agitation, aggression, sleep disturbances, wandering, and sexually inappropriate behaviors ^3^.

BPSD are common on inpatient psychogeriatric units (up to 90% of patients with diagnosed dementia) and are associated with a high economic burden ^4^. BPSD prevent patients with AD from living at home or in residential/nursing home settings ^3^. A strong neurobiological and clinical link has been demonstrated between cognitive dysfunction and neuropsychiatric symptoms in the pathophysiology of AD. BPSD are associated with accelerated disease progression ^5^. Recent studies show that they occur in an early phase of AD pathogenesis and are associated with greater amyloid deposition in the neurodegenerative process leading to AD ^6, 7^. Amyloid dysmetabolism and the accumulation of amyloid plaques, consisting of aggregates of a 42-amino acid peptide called amyloid beta (Aβ _1-42_), are two primary events in the pathophysiology of AD ^8, 9^. It is generally believed that oligomeric species of Aβ _1-42_ represent only the key initiator of a complex pathogenic cascade that includes microglia activation, hyperphosphorylation of tau protein, synaptic dysfunction, and finally neuronal death ^10^. According to this scenario, recent neuropathological studies suggest that BPSD are fundamental expressions of the underlying neurodegenerative brain disease in an early stage of AD and do not simply reflect the patient’s secondary response to his or her illness or just a behavioral product of diffuse brain disease ^11^. In particular, disruption of a cohesive noradrenergic locus coeruleus-thalamus linked system, owing to advanced neurodegeneration of locus coeruleus, has been proposed to lead to psychotic-like behavior in AD ^11^, in combination with an overactivation of dopamine D2 receptors in the mesolimbic pathway and hyperactive serotonin at 5-HT2A receptors in the cortex. Compared with patients with AD, those with psychosis of AD have shown significantly increased density of amyloid plaques and neurofibrillary tangles in the prosubiculum and middle frontal cortex and four/fivefold greater levels of abnormal tau protein in the entorhinal and temporal cortices ^11^. These neuropathological data can help explain epidemiological evidence where BPSD are more powerful predictors of incident mild cognitive impairment (MCI) than hippocampal atrophy ^12^ and can serve as predictors of progression from MCI to dementia, as demonstrated in two very large cohort studies ^5, 13^. In particular, the Cache County Dementia Progression Study, a longitudinal study of dementia progression conducted in 335 patients with incident AD, demonstrated that psychosis and agitation/aggression were associated with more rapid progression to severe dementia ^5^. A large cohort longitudinal study was performed in 1821 subjects with MCI and collected clinical data from 29 AD centers. Interestingly, this study found that neuropsychiatric symptoms such as depression and agitation may be among the earliest symptoms of preclinical stages of AD and were associated with a significantly increased risk of incident dementia and AD ^13^.

Recently, it was suggested that mild behavioral impairment, a neurobehavioral syndrome characterized by clinically relevant neuropsychiatric symptoms, represented a prodromal stage of AD and would be considered a novel target for future AD clinical trials and secondary-prevention strategies in patients with preclinical AD ^14^. Therefore, a deep understanding of the pathophysiology of BPSD in AD represents an essential step for the design of disease-modifying drugs able to counteract the progression of AD.

In this scenario, transgenic animal models of AD are an important tool to investigate the pathology underlying BPSD in human AD patients showing the early occurrence of behavioral symptoms in AD ^15^. In particular, animal models that exhibit the widespread amyloid or tau pathology such as 5xFAD (or both), human APP-overexpressing transgenic mice, or 3xTg-AD exhibit increased aggression in the resident/intrusion task relative to wild-type controls ^15^. In the near future, these animal models might help to identify new pharmacological targets for the treatment of specific BPSD such as aggression and agitation.

Unfortunately, current drugs for AD (three cholinesterase inhibitors and memantine) are officially approved in the EU for the treatment of cognitive deficits in AD, but none of these drugs has shown significant disease-modifying activity and clinically relevant efficacy against BPSD. The sole medication approved for the treatment of BPSD in AD (Canada and Europe only) is risperidone, which is limited to short-term treatment of aggression ^16^. First-generation antipsychotics, such as haloperidol, and second-generation antipsychotics, such as olanzapine and quetiapine, are commonly used off-label in the management of psychosis and agitation in AD patients despite a modest effect size and considerable safety concerns ^17, 18^.

A major goal in the treatment of BPSD in patients with AD is to reduce agitation (that is, the inability to stay calm, motor and verbal hyperactivity, and hyper-responsiveness until loss of control and aggressiveness) without inducing sedation in elderly patients ^19^. Sedation should be considered an adverse effect to be avoided in elderly patients with a high frailty index since it is associated with an increased cognitive impairment and an increased risk of falls and mortality ^20^. Frailty represents a limiting factor in elderly patients in the treatment of BPSD and can increase the vulnerability side effects of antipsychotics. Frailty can be either physical or psychological or a combination of the two ^21^. Frailty is associated with impaired cognitive performance, and the presence of physical frailty increases BPSD burden in patients with AD ^22^. According to this scenario, non‐pharmacological approaches are usually the first option when addressing BPSD in frail patients with AD, but when these are unsuccessful and risk of self‐harm or harm to others persists, pharmacological treatment is needed ^19^.

Current guidelines recommend the use of antipsychotic drugs, but only after first-line non-pharmacological interventions and for no longer than 12 weeks ^17, 23^ because the long-term use is associated with an increased risk of mortality and an increased frequency of cerebrovascular events in patients with dementia ^18, 24^. The long-term administration of antipsychotics is recommended only in patients with extremely severe initial manifestations of BPSD ^3, 18^.

Recent international consensus panels and algorithm-based approaches have suggested alternative pharmacological approaches when antipsychotics failed, such as the use of carbamazepine (neuroscience-based nomenclature [NbN]: glutamate: voltage-gated sodium and calcium channel blocker, step 3); citalopram (NbN: serotonin reuptake inhibitor, step 4), gabapentin (NbN: glutamate: voltage-gated calcium channel blocker, step 5), and prazosin (NbN: noradrenaline receptor antagonist, step 6), although they show limited effectiveness compared with second-generation antipsychotics ^19^. The algorithm also allows for the possibility of supplementing medication given in the main sequence with drugs. Interestingly, trazodone (NbN: serotonin receptor antagonist and receptor agonist) was selected as the drug of choice and can be used, for example, in combination with antipsychotics (it might help to reduce the dose of antipsychotics) or other drugs in the main algorithm sequence ^19^.

As discussed above, second-generation antipsychotics remain the mainstay for the treatment of agitation and aggression in patients with AD. A meta-analysis of 23 randomized controlled trials examined the clinical efficacy of second-generation antipsychotics in the treatment of BPSD in comparison with placebo and demonstrated that aripiprazole (overall BPSD symptoms) and risperidone (psychosis, agitation, and overall BPSD symptoms) performed better than placebo in reducing neuropsychiatric symptoms ^18^. A previous Agency for Healthcare Research and Quality (AHRQ) Comparative Effectiveness Review included olanzapine (agitation) among the most effective antipsychotics ^25^, whereas evidence from a meta-analysis shows that quetiapine, though commonly prescribed, has no effect on BPSD at the dosages commonly prescribed ^26^, showing an effectiveness for BPSD only at higher doses (100–200 mg/day), which may not be well tolerated ^23^. On the basis of this evidence, new antipsychotic drugs with improved efficacy and safety are needed as an alternative to current antipsychotic drugs for the treatment of agitation and psychosis in AD ^17^.

Much effort has been expended in the last five years to develop new drugs for the treatment of these BPSD in AD. In this commentary, we will briefly examine some of the most relevant advances in this field, focusing on the recent positive clinical evidence observed with two new antipsychotic drugs: brexpiprazole and pimavanserin.

## Brexipiprazole for the treatment of agitation in Alzheimer’s disease

Agitation is one of the most distressing neuropsychiatric symptoms in patients with dementia and it affects at least 50% of patients with AD ^27^. The deficit of the cholinergic system appears more severe in AD patients displaying agitation or aggression ^11, 28^. AD is also associated with widespread deficits of the serotoninergic system in the hippocampus and in the frontal lobe. The impairment of serotoninergic system can partially contribute to explain the onset of agitation and irritability in AD patients ^11, 29^; dopaminergic alterations have also been reported, and an increased dopaminergic cerebellar turnover is linked to physically agitated behavior ^30^.

According to the definition proposed by the Agitation Definition Workgroup of the International Psychogeriatric Association, agitation in patients with cognitive disorders or dementia is consistent with emotional distress, including excessive motor activity, verbal aggression, or physical aggression and causes excess disability with regard to interpersonal relationships, social functioning, or activities of daily living, not attributable to another disorder (psychiatric, medical, or substance-related) ^31^. The Cohen-Mansfield Agitation Inventory (CMAI) is a 29-item, clinician-rated, 7-point scale and is a well-validated psychometric tool that can be used to assess both aggressive and non-aggressive behavior in clinical trials ^32^. Currently, no drugs are approved by the European Medicine Agency (EMA) or the US Food and Drug Administration (FDA) with a specific indication for agitation in AD. Brexpiprazole is a novel third-generation antipsychotic that acts as a dopamine D2 partial agonist, a partial agonist at serotonin 5-HT1A receptor, an antagonist at serotonin 5-HT2A/5-HT2B, and noradrenaline α1B/α2C receptors ^33^. Brexpiprazole has recently been approved by the FDA for the treatment of schizophrenia ^34^ and as adjunctive treatment to antidepressants for the treatment of major depressive disorder ^35^. Interestingly, in 2019, two distinct phase III clinical trials, with a total of nearly 700 adult participants with AD, reported that brexpiprazole 2 mg/day had the potential to be an efficacious, safe, and well-tolerated treatment for agitation in patients with AD ^36^.

According to the new NbN, novel psychotropic drugs, including antipsychotics, can be classified on the basis of their pharmacology and mechanism of action in order to provide physicians with clearer alternatives than the Anatomical Therapeutic Chemical system when deciding the proper therapeutic strategy ^37^. The bases for NbN are first and foremost the pharmacological domains (that is, dopamine, serotonin, and so on) and modes of action (receptor antagonist, receptor partial agonist, reuptake inhibitor, and so on) ( https://nbn2r.com/). According to the NbN, brexpiprazole is a receptor partial agonist (D2, D3, 5-HT1A), receptor antagonist (5-HT2A/B, α1B/α2C). Interestingly, this drug also acts as an antagonist at histamine H1 receptor with a threefold higher affinity than aripiprazole ^38^. This pharmacodynamic property can probably help explain its clinically relevant efficacy against agitation in patients with AD.

The new evidence on brexpiprazole stems from two 12-week, randomized, double-blind, placebo-controlled, parallel-arm studies (NCT01862640 andNCT01922258) well conducted in AD patients with clinically relevant symptoms of agitation or aggression confirmed by a score of at least 4 on the Neuropsychiatric Inventory - Nursing Home version (NPI-NH) Agitation/Aggression domain ^36^. Study 1 investigated two fixed doses of brexpiprazole (2 mg/day and 1mg/day), whereas study 2 investigated flexibly dosed brexpiprazole (0.5−2 mg/day). The primary efficacy endpoint was the change from baseline to week 12 in CMAI total score. In the first study, brexpiprazole 2 mg/day demonstrated statistically significantly greater improvement in CMAI total scores from baseline to week 12 than placebo; in the second study, *post hoc* analyses showed that only patients who were titrated to brexpiprazole 2 mg/day at week 4 demonstrated superiority over matched placebo patients ^36^. Interestingly, brexpiprazole 0.5−2 mg was safe and well tolerated in patients with AD with mild or moderate treatment-emergent adverse events (headache, dizziness, and somnolence). These data suggest that the slow brexpiprazole titration schedule (4 weeks) adopted in these two studies may show both higher clinical efficacy against agitation and improved tolerability and safety profile compared with currently available second-generation antipsychotics drugs. Notably, these two trials included only patients with AD, excluding individuals with a history of stroke or vascular dementia, in contrast with past clinical trials with risperidone and olanzapine, conducted in highly heterogeneous groups of patients with dementia. Three different phase III clinical trials are underway to evaluate the long-term safety and clinical efficacy of brexpiprazole in AD patients with agitation (NCT03724942, NCT03594123, and NCT03545584). These studies will be relevant for fast approval from the FDA and EMA of brexpiprazole as a new antipsychotic drug for the treatment of agitation in AD.

## Pimavanserin for the treatment of psychosis in Alzheimer’s disease

Psychosis, defined by the emergence of delusions and hallucinations, is somewhat distinct from other BPSD and occurs in 30% of patients with AD ^17^. As discussed above, the neurobiology of psychosis in AD involves different neurotransmitter systems (dopamine, serotonine, acetylcholine, and noradrenaline) and is strictly linked to the severity of cognitive decline and to increased levels of tau phosphorylation in the frontal cortex of patients with AD ^39, 40^. Therefore, it is expected that future anti-tau disease-modifying drugs might exert a relevant clinical efficacy against both cognitive and neuropsychiatric symptoms in patients with AD ^17^.

Pimavanserin is a new 5-HT2A receptor acting drug that has been given market authorization for Parkinson’s disease psychosis (PDP) with recommended doses of 34 to 40 mg once daily ^41^. According to the NbN, pimavanserin is a receptor antagonist (5-HT2A, 5-HT2C). The FDA has termed it an inverse agonist but this conclusion is based on *in vitro* data ^42^, and recent studies suggest that pimavanserin functions primarily as an antagonist at 5-HT2A receptors ^43^. Selectivity for 5-HT2 receptors and sparing the dopamine post-synaptic receptors differentiate pimavanserin from other antipsychotic drugs currently used in PDP. Pimavanserin binds with very high affinity (Ki 0.087nM) to 5-HT2A and fivefold lower affinity (Ki 0.44nM) to 5-HT2C and shows negligible binding at 5-HT2B and dopaminergic D3 receptors ^43^. Recently, pimavanserin received a 100% agreement from an international consensus panel when considering emerging and experimental pharmacological treatments for psychosis ^44^. This preliminary positive evaluation stems not only from the evidence of efficacy and tolerability of this drug in PDP patients with cognitive impairment (NCT00477672, NCT00658567, and NCT01174004) ^41, 45^ but also from the relevant results observed in a phase II clinical study conducted in 181 AD patients with psychosis ^46^. The phase 3, double-blind, randomized, placebo-controlled study clearly demonstrated the efficacy of pimavanserin (34 mg once daily for 6 weeks) versus placebo in the treatment of hallucinations and delusions associated with PDP with a robust clinical effect (effect size: 0.50) assessed by the Parkinson’s disease–adapted scale for the assessment of positive symptoms (SAPS-PD) ^45^. Interestingly, a *post hoc* subgroup analysis conducted from this phase 3 study showed that pimavanserin exerted a more robust clinical effect (effect size: 0.99) in PDP patients with cognitive impairment ^41^.

The phase II clinical study conducted in patients with AD was a 12-week, randomized, double-blind, placebo-controlled, single-center study to assess the safety and efficacy of pimavanserin 34 mg once daily in 181 nursing home residents who had AD with psychosis ^46^. The primary endpoint was mean change from baseline at week 6 on the Neuropsychiatric Inventory-Nursing Home Version psychosis score (NPI-NH-PS), and AD patients with a score of at least 4 on either the hallucinations or delusions component or a combined hallucinations and delusions score of at least 6 on the NPI-NH-PS were recruited ^46^. At week 6 (but not at week 12), pimavanserin demonstrated a significant treatment effect versus placebo, including a treatment difference of −1.84 and a Cohen’s *d* effect size of 0.32, without inducing adverse effects on motor function or cognition. Interestingly, a recent subgroup analysis in patients with more severe psychosis at baseline (NPI-NH-PS ≥12) shows larger treatment effects (delta of−4.43 and Cohen’s *d* effect size of 0.73) with an at least 30% improvement of NPI-NH-PS in 88.9% of pimavanserin-treated patients versus 43.3% of placebo-treated patients ^47^. These results are particularly relevant when considering the low effect size (0.2) typically reported with second-generation antipsychotics ^48, 49^ and higher risk of mortality compared with placebo observed with these drugs ^50^. Furthermore, the tolerability profile of pimavanserin seems to be favorable with a rate of adverse events (urinary tract infection, fall, and agitation) similar between treatment groups ^47^, although the safety of the drug is being re-evaluated from May 2018 following reports of serious adverse events in patients with PDP ^51^. Pimavanserin can induce electrocardiographic QT-interval prolongation and is not recommended for patients with preexisting QT prolongation or a history of arrhythmias. Other pimavanserin-induced side effects include gait instability, falls, confusion, edema, and constipation. Further long-term studies are needed to better understand the safety profile of pimavanserin in patients with AD. In particular, a phase III, double-blind, placebo-controlled, relapse prevention study of pimavanserin for the treatment of hallucinations and delusions associated with dementia-related psychosis (DRP) (NCT03325556) is evaluating the efficacy and safety of pimavanserin in 360 individuals with DRP ( Table 1). The design of this study is interesting because it comprises a first period of 12 weeks of open-label treatment with a flexible dose of pimavanserin followed by blinded randomized withdrawal of treatment or continued pimavanserin therapy (20 or 34 mg) for 26 weeks ^41^. The study will be essential to validate the clinical efficacy of pimavanserin in the treatment of psychosis in different types of dementia, including not only AD but also vascular dementia, dementia with Lewy bodies, and frontotemporal dementia. Most importantly, this study will better define the long-term safety profile (38 weeks) in patients with DRP. The study was completed at the end of 2019 and results are awaited ( https://clinicaltrials.gov).

**Table 1.** Current status of clinical development of brexpiprazole and pimavanserin for the treatment of agitation and psychosis in Alzheimer’s disease.

Drug	Indication/Field	Phase of study of action	Duration	Sample size	Dosing	Main result or status or both
Brexpiprazole	Agitation	III Clinical trial: NCT01862640	12 weeks	433 AD subjects	2mg/day Fixed dose	Brexpiprazole demonstrated superior efficacy versus placebo as assessed by CMAI. Safe and well-tolerated
Brexpiprazole	Agitation	III Clinical trial: NCT01922258	12 weeks	270 AD subjects	Flexible dose in the range of 0.5–2 mg/day	Patients who were titrated to brexpiprazole 2 mg/day at week 4 demonstrated superiority versus matched placebo as assessed by CMAI.
Brexpiprazole	Agitation	III Clinical trials: NCT03724942, NCT03594123, NCT03620981	14 weeks 12 weeks 10 weeks	157 AD 250 AD 407 AD	1–2 mg 2–3 mg 1–2 mg	Ongoing To evaluate the long-term safety and clinical efficacy of brexpiprazole
Pimavanserin	Psychosis	II	12 weeks	181 AD	34 mg once daily	Large treatment effects in AD patients with more severe psychosis at baseline (NPI-NH- PS ≥12)
Pimavanserin	Psychosis	III Clinical trial: NCT03325556	38 weeks 12 weeks Open- label + 26 weeks Double- blind period	360 patients with DRP	20 mg or 34 mg once daily based on their open- label phase	Ongoing A long-term relapse prevention study of pimavanserin for the treatment of hallucinations and delusions associated with DRP

AD, Alzheimer’s disease; CMAI, Cohen-Mansfield Agitation Inventory; DRP, dementia-related psychosis; NPI-NH-PS, Neuropsychiatric Inventory-Nursing Home Version psychosis score

## Conclusions

Considerable effort has been expended in the last five years to develop new antipsychotic drugs for the treatment of agitation and psychosis in AD, two BPSD that are strongly linked to disease progression and severity of cognitive decline in patients with AD. Current long-term use of second-generation antipsychotics is associated with an increased risk of mortality and an increased frequency of cerebrovascular events. Therefore, new antipsychotic drugs with improved efficacy and safety are needed in this area.

As indicated in this report, brexpiprazole and pimavanserin are two new antipsychotics drugs that recently showed positive clinical evidence of efficacy for the treatment of agitation and psychosis in AD. Brexpiprazole is a receptor partial agonist (D2, D3, 5-HT1A), receptor antagonist (5-HT2A/B, norepinephrine α1B/α2C) that exerts relevant clinical efficacy for the treatment of agitation in AD patients with an improved tolerability and safety profile compared with currently available second-generation antipsychotics drugs. Pimavanserin is a receptor antagonist (5-HT2A, 5-HT2C) that has been given market authorization for psychosis in PDP. Recent phase II studies suggest the efficacy of this drug in AD patients with more severe psychosis, although further long-term studies are needed to better define the efficacy and long-term safety profile of pimavanserin (in particular, the risk of QT-interval prolongation) in the treatment of psychosis in AD.

## Abbreviations

5-HT, serotonin; Aβ, amyloid beta; AD, Alzheimer’s disease; BPSD, behavioral and psychological symptoms of dementia; CMAI, Cohen-Mansfield Agitation Inventory; DRP, dementia-related psychosis; EMA, European Medicine Agency; FDA, US Food and Drug Administration; MCI, mild cognitive impairment; NbN, neuroscience-based nomenclature; NPI-NH-PS, Neuropsychiatric Inventory-Nursing Home Version psychosis score; PDP, Parkinson’s disease psychosis
